# Relatives’ level of satisfaction with advanced cancer care in Greenland – a mixed methods study

**DOI:** 10.1080/22423982.2017.1335148

**Published:** 2017-06-14

**Authors:** Mikaela Augustussen, Lise Hounsgaard, Michael Lynge Pedersen, Per Sjøgren, Helle Timm

**Affiliations:** ^a^ Ilisimatusarfik, University of Greenland, Nuuk, Greenland; ^b^ Greenland Center for Health Research, Nuuk, Institute of Nursing and Health Science, University of Greenland, Nuuk, Greenland; ^c^ Institute of Nursing and Health Science, University of Greenland, Nuuk & OPEN, Department of Clinical Research, University of Southern Denmark, Odense, Denmark; ^d^ Palliative Research Group, Department of Oncology, Rigshospitalet – Copenhagen University Hospital, Copenhagen, Denmark; ^e^ REHPA, Danish Knowledge Centre for Rehabilitation and Palliative Care, University of Southern Denmark, Odense, Denmark

**Keywords:** Relatives, advanced cancer care, Greenland, rural and remote areas, satisfaction

## Abstract

Palliative cancer care in Greenland is provided by health professionals at local level, the national Queen Ingrid’s Hospital and at Rigshospitalet in Denmark. To improve and develop care for relatives of patients with advanced cancer, we conducted a mixed method study examining relatives’ level of satisfaction with care and treatment and their current main concerns. The aim was to investigate relatives’ level of satisfaction with advanced cancer care and bring to light their current main concerns. The FAMCARE-20 questionnaire was translated to Greenlandic and pilot tested. The questionnaire was supplemented by open-ended questions about relative’s current main concerns and analyzed with a phenomenological hermeneutical approach. Greenlandic patients with advanced cancer who were previously participating in a prospective study were asked if their closest adult relative would participate in the study. Telephone interviews were conducted and relatives responded to the questionnaire. A total of thirty-two relatives were contacted by telephone and 30 (94%) completed the FAMCARE-20 questionnaire and answered open-ended questions. The highest rate of satisfaction was with the availability of a hospital bed (66%) and relatives were the most dissatisfied with the lack of inclusion in decision making related to treatment and care (71%) and the length of time required to diagnose cancer (70%). Responses to the open-ended questions revealed that relatives faced challenges in gaining access to information from health professionals. They experienced a lack of security, worries about the future and a lack of support at home. The study showed a substantial level of dissatisfaction among relatives of patients with advanced cancer. We strongly recommend a focus on psychosocial care, more access to information and to include relatives in decision making and in the future planning of palliative care services. An assessment of relatives’ needs is essential to develop an adequate palliative care in a range of settings.

## Introduction

There is an increasing prevalence of cancer in the circumpolar population [1]; in Greenland there were 164 cases [2] in 2012 and, according to a journal audit, Greenlanders are diagnosed late in the disease trajectory [3]. Greenland is an island with an area over 2,000 km^2^ and 56,000 inhabitants. More than 90% of the population are Inuit and approximately one third live in the capital, Nuuk, while the rest live along the coast in towns, small settlements and villages [4].

Since 2011, palliative care in Greenland has been on the political agenda because a report published in that year concluded that treatment and care of patients with advanced cancer could be improved and needed to be upgraded [5]. Patients with advanced cancer often receive oncological treatment in Queen Ingrid’s Hospital (QIH) in Nuuk but many have to go to hospitals located in Denmark, mainly Rigshospitalet in Copenhagen. Therefore, oncological treatment demands the temporary relocation of patients, while relatives are mostly left at home. Although, depending on the severity of the disease, referring physicians can decide that authorities should cover travel and accommodation costs for an accompanying close relative.

If patients are treated at home in Greenland, the local health care center can provide treatment and care for patients and their families. However, the 2011 report concluded that there was a lack of guidelines for the support of patients and relatives and also poor cooperation between municipal services and the health care system, generally [3].

In recent years, the number of people living alone has increased and in 2016, it reached nearly 17,000 [6]. This has social consequences because relatives often constitute a major resource for patients during severe disease. Relatives of patients with advanced cancer are heavily burdened because they face a life-threatening disease, and the level of support required by both patients and relatives can increase greatly as the disease progresses.

No studies from Greenland have been published to date concerning the satisfaction of relatives with cancer treatment and care. However, the need for more information has been documented as very important in addressing relatives’ needs in rural and remote settings in both Australia and Africa [7, 8]. Furthermore, in the US, a randomised controlled trial demonstrated that adequate support and therapy for families of patients with advanced cancer reduced the severity of complicated grief and the development of prolonged grief disorder [9].

Home is the preferred place of care and death for the majority of patients with advanced cancer [10]; however, particularly in rural and remote areas, it can be a drain on resources of informal family caregivers. A qualitative study among relatives in rural and remote settings in South Africa demonstrated a lack of access to information, which reduced families’ ability to cope with their situation [11]. Similarly, a study from Western Australia, documented that health care professionals faced challenges in not only delivering treatment and care to patients, but also in providing bereavement support for the relatives [12].

Support for the family during a severe and potentially fatal disease trajectory is, therefore, an important issue. Relatives’ level of satisfaction with cancer care is an important outcome measure [13], which until now has not been investigated in the Arctic area. Studies of relatives’ level of satisfaction with cancer care can be used to ensure care focuses on the needs of the whole family, with a view to enhancing support for the patient. This seems to be of particular relevance and importance in rural and remote areas since limited access to health care and lack of resources are well known challenges [14].

Therefore, the aim of the present study was to assess relatives’ level of satisfaction with advanced cancer care and to bring to light their current main concerns.

## Materials and methods

### Design

A population-based, cross-sectional survey with a mixed method approach was undertaken. The Greenlandic version of the FAMCARE-20 questionnaire was used, but supplemented with additional questions about the relatives’ current main concerns.

### Study setting

The study took place in Nuuk. Relatives were located in different parts of Greenland, so for geographical and logistical reasons questionnaires were primarily completed by telephone interview while relatives were at home.

### Participants and recruitment

Participants were recruited through patients with advanced cancer who had previously participated in a prospective study to identify their closest relative. Relatives were then contacted by telephone and asked to participate in the study.

### FAMCARE-20 questionnaire

The FAMCARE-20 questionnaire, which is based on a Likert scale, comprised 20 questions and was developed to assess families satisfaction with advanced cancer care. It had a conceptual structure, with four sub-scales that include: Five items about information from health care professionals, four items about availability of nurses and doctors, four items about psychosocial care and seven items regarding symptom control [15]. Scores range from one to five, where one reflects the highest level of satisfaction.

It was translated into Greenlandic by forward and backward translation and pilot tested by five relatives.

### Statistical analysis

Descriptive statistics of prevalence, mean and range for each item in the FAMCARE-20 questionnaire were calculated using SAS statistical software version 9.4.

Comparisons of satisfaction levels between categories “spouse/cohabiting partners” and “children/sibling/other” were conducted by applying the Wilcoxon test.

### Open-ended questions

The FAMCARE-20 questionnaire was supplemented with open-ended questions about the relatives’ main concerns regarding their situation at the time of the interview. Notes regarding relatives’ responses to the open-ended questions were taken during the interviews and documented in REDCap [16], which is a secure, internet-based documentation software developed for research. Finally, three narratives illustrating the situation and concerns of the relatives were added.

### Qualitative analysis

Interview text from the open-ended questions was interpreted and analysed with inspiration from philosopher Paul Ricoeur’s phenomenological-hermeneutical approach [17,18]. We had short narratives from relatives in note form and the approach made it possible to analyse and interpret the entire material as one text. The intention was to describe the text and interpret the narratives from the relatives and thereby derive common themes.

The interpretation was carried out in three steps: naïve reading, structural analysis and comprehensive understanding/critical in-depth interpretation [18]. The naïve reading was an open-minded process using a phenomenological approach and all notes from the interviews were read repeatedly to get a sense of the text as a whole. Structural analysis is a dialectical process between naïve reading and the interpretation of the text, intended to identify units of meaning (what is said) and units of significance (what the text is talking about). This level of the analysis led to an identification of the main themes regarding being a relative of a patient with advanced cancer in Greenland. Narratives were then inferred to describe common issues and problems – which constitutes the critical, in-depth interpretation element of the analysis.

### Ethics

The project was assessed according to the Helsinki declaration and approved by the Ethics Committee for Medical Research in Greenland (2014-102760) and the Data Protection Agency in Denmark (2014-41-3660). Informed consent was obtained from all individual participants included in the study.

## Results

### Response rate

Between July 2015 and March 2016, a total of 58 patients receiving treatment in the Greenlandic health care system participated in the prospective study, and we ended up with 32 relatives, all of which were contacted by telephone.

Two did not respond; a total of 30 relatives, corresponding to 94% (30/32), were willing to participate. Three of the relatives chose to fill in the questionnaire and return it by post, while the rest [19] were interviewed by telephone.

### Characteristics of the participants

Approximately 80% of the respondents lived in small villages and settlements located along the coast of Greenland; 10% lived in other villages and 10% were residents of Nuuk. Sixteen relatives lived together with the patient, and 14 did not.

Characteristics of the relatives are shown in Table 1. The mean age of participants was 48. Thirteen were spouses or cohabiting partners, while 17 were grown-up children or siblings. Twenty-seven were females and only three were males.

**Table 1.** Characteristics of participants.

Variable	n=30
Mean age, years	48
Spouse/cohabiting partner	13
Children/sibling/other	17
Males	3
Females	27

### Satisfaction with treatment and care

Table 2 shows the overall proportions of satisfaction with treatment and care. In general, relatives were most satisfied with the availability of hospital beds and the length of time required to treat symptoms and provide pain relief. However, relatives often choose the “undecided” category and three respondents were unable to respond to the entire questionnaire due to limited insight into the treatment and care provided.

**Table 2.** Responses to the questionnaire reported as percentages.

Item number	n=	Mean scores	Very satisfied (%)	Satisfied (%)	Undecided (%)	Dissatisfied (%)	Very dissatisfied (%)
1. The patient’s pain relief	30	2.6	6.7	56.7	10	26.7	0
2. Information provided about the patient’s prognosis	29	3.2	6.9	24.1	20.7	31.0	17.2
3. Answers from health professionals	30	2.6	13.3	40	26.7	13.3	6.7
4. Information given about side effects	29	2.9	3.5	48.3	13.8	27.6	6.9
5. Referrals to specialists	29	2.9	6.9	44.8	13.8	20.7	13.8
6. Availability of hospital beds	29	2.4	20.7	44.8	13.8	13.8	6.9
7. Family conferences held to discuss patient’s illness	29	3.4	3.6	25	21.4	23	17.7
8. Speed with which symptoms are treated	28	2.5	14.3	50	10.7	17.9	7.1
9. Doctors’ attention to patient’s description of symptoms	28	2.8	7.1	42.9	32.1	3.6	14.3
10. The way tests and treatments are performed	29	2.7	10.3	51.7	10.3	17.2	10.3
11. Availability of doctors to the family	29	3.1	3.5	37.9	10.3	34.5	13.8
12. Availability of nurses to the family	28	3.1	7.1	25	32.1	21.4	14.3
13. Coordination of care	28	2.6	14.3	42.9	17.9	17.9	7.1
14. Time required to make a diagnosis	27	3.8	7.4	18.5	3.7	25.3	44.4
15. The way the family is included in treatment and care decisions	28	3.8	3.6	17.9	7.1	39.3	32.1
16. Information given about how to manage the patient’s pain	27	3.2	3.7	29.6	29.6	18.5	18.5
17. Information given about the patient’s tests	27	2.9	3.7	51.9	7.4	22.2	14.8
18. How thoroughly doctors assess the patient’s symptoms	27	2.9	3.7	40.7	25.9	22.2	7.4
19. The way tests and treatments are followed up by the doctor	28	2.9	3.6	46.4	21.4	17.9	10.7
20. Availability of the doctor to the patient	27	2.7	11.1	44.4	11.1	29.6	3.7

Relatives were most undecided regarding the availability of nurses to the family (32%), the doctors’ attention to the patient’s description of symptoms (32%) and the information given regarding management of patient’s pain relief (30%).

### Dissatisfaction with treatment and care

The most pronounced dissatisfaction was related to the time required to make a diagnosis and to families involvement in treatment and care decisions.

Proportions in Table 2 are based on the “dissatisfied” or “very dissatisfied” responses, and are presented in ranked order according to four subthemes: information given, availability of treatment and care, physical patient care and psychosocial care, all of which are elaborated below.

#### Information given

The items with the highest proportion of “dissatisfied” or “very dissatisfied” responses concerned the information given about the patient’s prognosis (48%), information given about pain management and patients’ tests (37%), information given aboutside effects (35%) and answers from health care professionals (20%).

#### Availability of treatment and care

The items with the highest proportion of “dissatisfied” or “very dissatisfied” responses concerned the availability of doctors to the family (48%), availability of the nurses to the family (35%), availability of the doctor to the patient (33%) and availability of a hospital bed (21%).

#### Physical patient care

The highest proportion of “dissatisfied” or “very dissatisfied” responses concerned the length of time required to get a diagnosis (70%), referrals to specialists (34%), doctors’ assessments of patients’ symptoms (30%), the way tests were followed up by the doctor (29%), the way tests and treatments were performed (27%), patients’ pain relief (27%) and the time required to treat symptoms (25%).

#### Psychosocial care

The highest proportion of “dissatisfied” or “very dissatisfied” responses concerned the way the family was included in treatment and care decisions (71%), family conferences held to discuss the patient’s illness (41%), coordination of care (25%) and doctors’ attention to the patient’s descriptions of symptoms (18%).

Comparisons of means regarding the level of satisfaction between “spouse/cohabiting partners” and “children/sibling/other” showed no statistical differences with p-values >0.05.

### Results of the qualitative analysis

The structural analysis showed that participants talked about being a relative of a patient with advanced cancer who was being treated elsewhere, their experience of lack of information about the disease and about having to manage all the practical work at home (Table 3).

**Table 3.** Structural analysis.

Units of meaning “What is said”	Units of significance: “What the text is talking about”	Main themes
“My husband is the one doing all the practical things at home, I dread the day it’s me alone”, wife of a patient with advanced cancer	Fear of losing the loved one Suffer deprivation when the patient is travelling for treatment	Theme 1: to be relative of a patient with advanced cancer who is being treated elsewhere
“I miss my sister when she goes for treatment in another place – I help her all I can, also financially”, sister of cancer patient	Inability to accompany the sick person during treatment	
“I think it’s hard when my sick father has to travel so far to get treatment. It might be safer for everyone if relatives could travel with them, but there are no resources”, son of a patient with advanced cancer		
“I have a need to be with my husband while he is talking with the doctors”, wife of a patient with advanced cancer	The need to be involved during treatment	Theme 2: lack of information about the disease
“I fear that there is more wrong than what my husband tells me”, wife of a cancer patient	Uncertainty and anxiety about the prognosis of the disease	
“I need to talk to the doctors about what the purpose of the treatment is and what the plans are for my husband”, wife of a patient	Lack of understanding of purpose of the treatment	
“What if you have a job and you also need to take care of everything at home?”, wife of a man with advanced cancer	Having to take responsibility for the home and take care of the sick person at home	Theme 3: having to manage all the practical work at home
“I do not feel I can help my sister with her medicine because I know nothing about it”, sister of a woman with advanced cancer	Lack of knowledge of medicine administration	
“I think he gets too little help when he is so sick. I help him with all the practical things at home”, close friend of a man with advanced cancer living alone in his own home	Lack of support and help at home	

Three narratives were derived from the interpretation which illustrate the challenges involved in being a relative of a patient with advanced cancer in Greenland (Table 4). Firstly, it can be difficult to follow the trajectory of the disease because treatment and care often take place far away, which leads to a lack of information and anxiety about the situation. The emotional burden of being separated while the patient is treated away from home was apparent and it also became clear that the lack of information and consequences of being uninformed about the diagnosis and prognosis of the advanced cancer were experienced as a burden.

**Table 4.** Narratives.

	The course of the disease	The need for help at home	Communication with the health care system	Main concerns
Erika and Sven	Erika, 41 years old, is the daughter of Sven. Sven is 64 years old and was diagnosed with cancer 15 years ago. Back then, he received treatment in Denmark with radiation and chemotherapy. Erika did not have the opportunity to be with him during treatment. Sven had a relapse of cancer a year ago and since then he has lived in a small settlement. He needs to travel every third week to QIH for chemotherapeutic treatment.	Erika sees that her father’s condition has deteriorated and he is no longer capable of fishing,hunting and providing his family with meat. Erika helps with the cleaning, and fetching and carrying water, because Sven lives in a house without running water. What she considers to be a difficult task is the administration of her father’s medicine, particularly analgesics.	Erika has a little contact with the local health worker, but when her father gets in pain, is nauseous or constipated or what she perceives as a serious problem – lack of appetite – she doesn’t know where or how to get in contact with other health care professionals. Erika has many thoughts about the disease and doesn’t know if her father will recover.	Erika is comfortable with the father being able to stay in his own home town, but she is uncertain if this can continue. She has a lot of speculations about what will happen in the near future. The father has requested employment and support allowance, but has not yet received it. Erika helps her father economically.
				There are no opportunities to get advice and guidance on their situation in their village. Sven has given Erika the telephone number to QIH, but Erika has not yet contacted them.
Ole and Debora	Ole has been married to Debora for 45 years. They are both 68 years old. Debora got cancer in 2016 and was diagnosed at a late stage. She was given the opportunity to receive palliative chemotherapy, which she gets in her small hometown every third week.	Both Debora and Ole have difficulties in managing the practical work in the house and receive help from local municipal workers. A nurse comes regularly to administer Debora’s medicine and in collaboration with Debora and Ole she can contact the local hospital.	Ole always goes with Debora for her treatment and he knows about the plan for treatment. Their adult children have also been participating in conversations with the doctors and nurses. They know who to contact if they have doubts and Ole (as a relative) can stay in a bed at the hospital, if he wants to stay with Debora when she is hospitalised.	At the hospital, Ole meets other relatives in a similar situation and appreciates that kind of contact with others. Besides that, they often go to church and the couple also has contact with the priest if they need to talk. The community is small and everybody knows each other. Sometimes Ole needs to talk to someone who does not know them. The big concern for Ole is the day when he will be left alone.
Else and Aron	Else is 36 years old. Her father, Aron, at 69, has had a cancer diagnosis for the last six years and his treatment demands that he relocate to Denmark. Since then Aron has travelled long distances and the last decision the physicians made was that they could offer life-prolonging treatment in Denmark.	When Aron is at home, he gets help from Else and her family. But he has only been home for limited periods, primarily for holidays.	Else has not been involved in communication with the health care professionals at any time during her father’s disease. But from what her father has told her, she comes to realise that her father suffers from incurable cancer.	Else calls Aron every day to hear how he is doing and she can hear her father’s condition is worsening. Else has no knowledge of how long her father will be gone for treatment. The local health workers have not received any information yet. Else would like to be with Aron.

Furthermore, relatives expressed how uncertainty about the future and their unawareness of the patient’s treatment and care plan had a negative impact on their everyday life. Having to manage all the practical work at home and the informal care, e.g. cleaning, medicine administration or other practical issues, while their loved one was at home was a big challenge.

The narratives illustrate some common and typical issues experienced by relatives in relation to the trajectory of the disease, the practical needs at home and relative’s limited or lack of contact with the health care system, together with a range of other factors that influenced their situation. Geographical factors have a great impact on access to information and the narratives also reflected differences in access to practical help and contact with health care professionals. The narratives are intended to illustrate the individual stories behind the (dis-)satisfaction data, and the themes of concern, such as being separated, being uninformed and bearing the burden alone as the nearest relative.

## Discussion

The aims of the study were to measure relatives’ levels of satisfaction with the care of Greenlandic advanced cancer patients and furthermore, to explore relatives’ main concerns at the time of the interview. This is the first attempt to assess relatives’ perspectives on the advanced cancer care of Greenlandic patients.

The overall finding is that relatives face multiple problems in supporting family members with advanced cancer. In general, relatives have limited access to formal services and limited or no contact with health care professionals. This causes insecurity, uncertainty and worry regarding prognosis and the aims of treatment and care. Overall, the present study revealed a great deal of dissatisfaction on the part of these informal caregivers.

The most pronounced dissatisfaction, categorised as “very dissatisfied”, was associated with the delay in getting a diagnosis and the lack of inclusion in decision making regarding the care and treatment of loved ones. The highest degree of satisfaction, categorised as “very satisfied”, concerned the availability of a hospital bed and the establishment of pain mangement.

The Greenlandic government has published several documents that lay out plans and goals for the future treatment and care of patients with cancer [3,5,20], but relatives are scarcely mentioned. According to the WHO’s definition of palliative care, supportive care for relatives is essential in the provision of palliative care, and the whole family should be the target in a holistic approach. Professional support for relatives is substantial for informal caregivers, who are at risk of experiencing complicated grief and depression post-loss [21]. Thus, various interventions are identified to support informal caregivers [22].

Strikingly, our study showed that the dissatisfaction of Greenlandic relatives was much higher than relatives in Denmark and Norway [13,23]. We found that 71% of the relatives reported being “dissatisfied” or “very dissatisfied” with the lack of inclusion in treatment and care decisions and that 70% were “dissatisfied” or “very dissatisfied” with the time required to make a diagnosis. The corresponding figures in a Danish study were 14% and 28%, respectively, and in a Norwegian study 38% and 15%, respectively. This could be explained by several reasons related to geography and the structure of the health care system in general: Firstly, health care services are limited and sometimes absent from remote areas, and secondly some patients have to travel for treatment and care, leaving relatives behind where they can get excluded from professional treatment and communication. These findings could also be linked to Greenlands small population. However, it is well known that an extremely negative event can trigger dissatisfaction [24], which in turn can explain the pronounced dissatisfaction with the time required to receive a diagnosis and the distress this can cause. In addition, health issues among indigenous people can face challenges related to social life [25] and the Greenlandic population is thus also affected by these challenges. These findings reveal important issues, such as communication between health care professionals and the needs of families who are facing serious illness.

Satisfaction as a single outcome is not enough to fully express the relatives’ complex and emotional situation [26]. We consider that our findings in the analysis of the open-ended questions highlight important issues that should be addressed in the future planning of palliative care. Our small quantitative dataset is supported by the relatives’ responses to the open-ended questions, which stated they had few opportunities to receive adequate information and little contact with health care professionals, apart from limited access to formal care services.

A previous study showed that informational needs of relatives residing in remote and rural settings differed from those in urban areas and, therefore, individually-tailored information is necessary in order to meet the needs of the family [27]. Besides informational needs, other factors, such as transportation and practical issues, are well-known barriers for people living in remote and rural areas [19]. This is in line with our findings, because the majority of relatives lived in areas outside Nuuk, so without direct access to the department of oncology at QIH. A qualitative study of relatives of patients in rural and remote areas who travelled for palliative treatment showed that relatives were burdened with an enormous responsibility for their loved ones [28]. Therefore, we strongly recommend more inclusion of relatives in care and treatment, regardless of the fact that infrastructural and geographical conditions can be barriers.

This study has limitations. Because the quantitative dataset was small, we were only able to perform simple statistical analysis with prevalence and mean scores. However, we do consider our study to be representative of the population of relatives of Greenlandic patients with advanced cancer. A total of 58 patients participated in the prospective study and we intended to have an equivalent number of relatives. The main reason for the reduced number of relatives was that we had originally chosen a different questionnaire, which turned out not to be suitable in a Greenlandic context and therefore was not included in the present study. Therefore, we ended up with fewer participants using the FAMCARE questionnaire. In addition, the relatives had to evaluate different settings in Greenland and some had difficulty assessing the treatment and care if the patient had received treatment in both Denmark and Greenland. Furthermore, the category “undecided” can be difficult to interpret, but by replacing this category as a missing value the means were nearly the same. It could be assumed, however, that the relatively substantial element of “undecided” responses was linked (in its own right) with the lack of information about the disease trajectory, which is pointed out by the respondents.

### Conclusions

In conclusion, this study shows substantial dissatisfaction among relatives of patients with advanced cancer. The study also illustrates the special situation of relatives of cancer patients in Greenland. Relatives are a major resource for patients with advanced cancer during treatment and at end of life. Implications of the study are that health care professionals need to increase their level of support and amount and type of information given to relatives and include relatives more in the care and treatment. The FAMCARE questionnaire, supplemented with open-ended questions, was useful in the assessment of unmet needs, but further studies are needed to properly validate the questionnaire.
